# Pathological Mechanisms in Diabetes of the Exocrine Pancreas: What’s Known and What’s to Know

**DOI:** 10.3389/fphys.2020.570276

**Published:** 2020-10-28

**Authors:** Qiong Wei, Liang Qi, Hao Lin, Dechen Liu, Xiangyun Zhu, Yu Dai, Richard T. Waldron, Aurelia Lugea, Mark O. Goodarzi, Stephen J. Pandol, Ling Li

**Affiliations:** ^1^Department of Endocrinology, ZhongDa Hospital, School of Medicine, Southeast University, Nanjing, China; ^2^Institute of Pancreas, Southeast University, Nanjing, China; ^3^Department of Clinical Science and Research, ZhongDa Hospital, Southeast University, Nanjing, China; ^4^Nanjing Foreign Language School, Nanjing, China; ^5^Division of Gastroenterology, Department of Medicine, Cedars-Sinai Medical Center, Los Angeles, CA, United States; ^6^Division of Endocrinology, Diabetes and Metabolism, Department of Medicine, Cedars-Sinai Medical Center, Los Angeles, CA, United States

**Keywords:** endocrine, exocrine pancreas, diabetes, chronic pancreatitis, pathological mechanisms

## Abstract

The clinical significance of diabetes arising in the setting of pancreatic disease (also known as diabetes of the exocrine pancreas, DEP) has drawn more attention in recent years. However, significant improvements still need to be made in the recognition, diagnosis and treatment of the disorder, and in the knowledge of the pathological mechanisms. The clinical course of DEP is different from type 1 diabetes mellitus (T1DM) and type 2 diabetes mellitus (T2DM). DEP develops in patients with previous existing exocrine pancreatic disorders which damage both exocrine and endocrine parts of pancreas, and lead to pancreas exocrine insufficiency (PEI) and malnutrition. Therefore, damage in various exocrine and endocrine cell types participating in glucose metabolism regulation likely contribute to the development of DEP. Due to the limited amount of clinical and experimental studies, the pathological mechanism of DEP is poorly defined. In fact, it still not entirely clear whether DEP represents a distinct pathologic entity or is a form of T2DM arising when β cell failure is accelerated by pancreatic disease. In this review, we include findings from related studies in T1DM and T2DM to highlight potential pathological mechanisms involved in initiation and progression of DEP, and to provide directions for future research studies.

## Introduction: Clinical Features of DEP

Diabetes of the exocrine pancreas (DEP),refers to diabetes mellitus (DM) secondary to various exocrine pancreatic diseases such as pancreatitis, trauma/pancreatectomy, pancreatic neoplasia, etc. Due to confusion with type 2 diabetes mellitus (T2DM) (Petrov, 2017), it has been underestimated in clinical practice for a long time. The incidence of DEP varies with geographical distribution and etiology (Ewald and Hardt, 2013; Pendharkar et al., 2017; Woodmansey et al., 2017; Bharmal et al., 2020a), with estimated true prevalence ranging from 1 to 9% of all diabetic patients (Hart et al., 2016). It has been noted that compared to other DM patients, the DEP patients present additional symptoms related to pancreatic disease, including decreased glucagon and somatostatin, pancreatic exocrine insufficiency (PEI), malabsorption of nutrients and micronutrients, severe and painful gastrointestinal symptoms, and nutritional deficiencies (Wynne et al., 2019). These additional morbidities promote greater blood glucose fluctuations. Although there have been contradictory observations about glycemic control in DEP (Jethwa et al., 2006; Ewald et al., 2012), it is generally believed that serum glucose levels in DEP are difficult to control, with alternately occurrence of hypoglycemia and hyperglycemia, (also known as “brittle diabetes”) (Larsen et al., 1987; Larsen, 1993).

Currently, there is no uniformly recognized diagnostic criterion for DEP. Identification of the disease emphasizes the detection of exocrine pancreatic disorders and pathological changes of the exocrine pancreas different than the autoimmune mechanism which is the characteristic of type 1 diabetes mellitus (T1DM). The current proposed diagnostic criteria include evidence of pancreatic exocrine disease such as functional insufficiency (identified with tests of the fecal elastase-1 or exocrine pancreatic function) and pathological changes of pancreatic imaging [computerized tomography (CT), magnetic resonance imaging (MRI), endoscopic ultrasound], as well as potential alterations in incretin secretion and decreased levels of fat soluble vitamins (A,D,E, and K) in serum (Ewald and Bretzel, 2013; Wynne et al., 2019). In addition, certain novel biomarkers showed potential in discriminate DEP from other diabetes (Olesen et al., 2019; Bharmal et al., 2020b; Gold-Smith et al., 2020).

Similar to other DMs, the major goal of DEP treatments is to reduce hemoglobin A1c (HbA1c) levels for minimizing the macrovascular and microvascular complications risk, as well as delay the development of pancreatic cancer. Traditionally, physicians tend to choose similar drugs used to treat T2DM (Rickels et al., 2013), however recent knowledge about the potential differences from other diabetes suggested more careful treatment strategy. Lifestyle adjustment and dietary interventions are critical to improve nutrition status and overall health outcomes. As for the anti-hyperglycemic medication, insulin therapy is preferred for most patients due to common insulin deficiency in DEP patients. In addition, insulin was suggested to benefits nutrition of patients. However, in considering of preserved peripheral insulin sensitivity, the dosing should be titrated, similar as in T1DM. Metformin was suggested to be used in patients with DEP due to chronic pancreatitis (CP), owe to reduced risk of pancreatic ductal adenocarcinoma (PDAC) (Gudipaty and Rickels, 2015; Wynne et al., 2019). Due to potential differences in symptoms and etiology between DMs, personalized hyperglycemia solutions should pay special attention to drug indications and contraindications. For example, although being preferred selections for DM treatment in many situations, incretin-based therapies such as glucagon-like peptide-1 (GLP-1) receptor agonists have been suggested to be associated with increased pancreatitis risk (Buse et al., 2017; Abd El Aziz et al., 2020). Although more thorough studies have suggested that incretin-based treatment did not increase pancreatitis risk (Wang et al., 2015, 2018; Abd El Aziz et al., 2020), it is still suggested to be used with vigilance (Buse et al., 2017; Abd El Aziz et al., 2020). Therefore, application of these drugs in DEP patients should be done with caution (Alves et al., 2012). Moreover, DM treatments in these patients should be applied in combination with pancreatic enzyme replacement therapy (PERT) for PEI and proper diet to maintain nutritional requirements and the absorption of fat-soluble vitamins (Duggan and Conlon, 2013; Lohr et al., 2017).

## Etiology and Pathology of DEP

While CP was originally thought to be the most common cause of DEP, the greater numbers of patients with acute pancreatitis (AP) led to recent recognition that 80% of pancreatitis-related DEP is due to AP and 20% to CP (Petrov and Yadav, 2019). The pancreas is composed of both exocrine and endocrine structures. In addition, the endocrine part of the pancreas contains five different types of cells (α, β, δ, PP, and ε). The endocrine hormones secreted include glucagon, insulin, somatostatin, pancreatic polypeptide and ghrelin (Da Silva Xavier, 2018; El Sayed and Mukherjee, 2019). Disorders of the exocrine compartment including AP and CP, pancreatic tumor, pancreatic trauma, cystic fibrosis, partial pancreatectomy, hemochromatosis, and pancreatic agenesis precede are characteristics of DEP, with the most common co-existing disorder being pancreatitis (Abu-Bakare et al., 1986; Expert Committee on the Diagnosis and Classification of Diabetes Mellitus, 2003; Ewald et al., 2012; American Diabetes Association, 2019). As a result of exocrine pancreatic disease, DEP is featured with pancreatic tissue damage compromising exocrine cells and cell subtypes within the islets of Langerhans (Walling and Freelove, 2017; Hammad et al., 2018; Pham and Forsmark, 2018). In this respect, DEP is significantly different from T1DM, in which autoimmune damage of β cells and secondary inflammation cause relatively limited damage to the exocrine compartment.

While much research indicates that extensive destruction of islet cells is the main reason of the development of DEP from CP (Ewald et al., 2012), β cell dysfunction without extensive destruction has also been observed in CP (Sasikala et al., 2012), but the contribution of other endocrine and exocrine cell types to the pathogenesis of DEP is unclear.

Besides cellular damage and dysfunction of pancreatic cells, other factors such as PEI and malnutrition, which are common in DEP, may promote DM pathology through mechanisms involving regulation of incretin secretion and perturbing intestine-pancreas crosstalk. These factors should be addressed in completing our understanding of DEP pathogenesis.

Since research studies specific for DEP are still rare, the understanding of DEP pathology often relies on the current knowledge about the characteristics and mechanisms underlying T1DM and T2DM pathology. It is often the differences in the extensities and combinations of these basic pathological changes that separate different DMs. In the next sections, we review different potential mechanisms involved in the development of DEP.

## Contribution of Changes in β Cell Mass and Function to DEP Pathology

β cells and insulin are the central players in DM development. The β mass, referring to the total number of β cells, as well as the functional status of these cells, are the major determinant of plasma insulin levels. Reduced cell mass or function can both lead to insufficient insulin levels, which can result in hyperglycemia and diabetes. In T1DM, loss of β cell mass, as the result of autoimmune damage of β cells, is the main cause of disease. In most cases of T2DM, insulin resistance is the initial pathological mechanism, with hyperglycemia developing when β cells fail to compensate with insulin hypersecretion. Dysfunction of β cells also plays critical roles in T1DM and T2DM. In T1DM, functional exhaustion and degranulation have been noticed in the remaining β cells after long disease period (Rojas et al., 2018). In T2DM, β cell compensation is characterized by enhanced insulin secretion and β cell hyperplasia, hypertrophy, and increased proliferation. But chronic hyperglycemia will eventually result in β cell exhaustion, the reversal of β cell structural adaptations evident by hypoplasia, hypotrophy, reduced proliferation, and dedifferentiation, all resulting in insulin hyposecretion (Chen et al., 2017). In addition, dynamics of insulin release may be changed at late stages, presented as damaged response in the first phase, as well as destruction of conventional oscillation release mode (Del Prato et al., 2002). It seemed that changes in cell functions play more important role than cell mass alteration in this process.

Many cases of DEP caused by CP are likely due to the β cell mass loss and insulin secretion reduction. It has been reported that β cell mass was reduced by approximately 29% in CP patients; and that reductions in insulin secretion and hyperglycemia occur in CP patients with β cell mass < 40% of the normal (Meier and Giese, 2015). Damages in functional islet mass, and correlated early-phase insulin secretion, was reported in CP patients with early development of postprandial hyperglycemia (Sheikh et al., 2017). In CP patients, β cell function impairment and decrease in exocrine pancreatic function often co-exist. It has been reported that insulin secretion is closely correlated with pancreatic enzyme stimulation. Moreover, in patients with cystic fibrosis, it was noticed that reduced β-cell secretion capacity was only detected when pancreatic exocrine secretion was insufficient (Nyirjesy et al., 2018).

How important a role that β cell mass and functional changes play in the pathogenesis of DEP is still unclear, mainly because of the rarity of human samples. In addition, technologies to investigate human β cell mass need to be further perfected. It has been suggested that impairment of β cell function may occur at early CP stages, while only when significant fibrosis appears that clinical diabetes symptoms will present (Sasikala et al., 2012). Tissue damage to the pancreas in CP showed significant specificity to the exocrine compartment and has little effect on the endocrine islets, as supported by the non-significant turnover of β cells although the number of apoptotic acinar cells increased about 10 times (Schrader et al., 2009). In addition, β cell destruction appears to be slower and its function better preserved in CP patients than in comparable T1DM patients. In sum, the evidence suggests that similarly to T1DM and T2DM, dysregulation of insulin secretion function in the β cell is an early pathological event in DEP. Loss of total β cell mass is also critical in disease progress, but the exact level of β cell mass reduction required for disease manifestation has not been determined yet.

## Cell Death and Inflammation as Potential Drivers of β Cell Mass Loss and Dysfunction in DEP

Various stimulations in diabetes may cause β cell death, including inflammatory and immune responses, stress factors such as reactive oxygen species (ROS), endoplasmic reticulum (ER) stress, as well as damages in cell components [such as deoxyribonucleic acid (DNA) fragmentation] and functions such as excessive islet amyloid. The mechanism underlying most cell death process, such as autophagy, inflammasome activation and mitochondrial dysfunction also play important role in β cell death (Rojas et al., 2018). In T1DM, β cell destruction is induced by an ongoing immune cell infiltration, and is aggravated through ER stress and apoptosis which are caused by disturbed glycemic metabolism (Kurrer et al., 1997). In T2DM patients, the processes underlying β cell mass loss are unclear but likely involve increases in β cell apoptosis due to insulin resistance and subsequent β cell exhaustion.

Inflammation and oxidative stress play critical roles in reduced β cell mass in both T1DM and T2DM (Imai et al., 2016; Halim and Halim, 2019; Tsalamandris et al., 2019). Macrophage-mediated inflammatory responses within islets were suggested as major contributors of reduced β cell survival and function. However, recent studies indicate that macrophages can protect β cells during pancreatitis and support β cell proliferation and regeneration (Van Gassen et al., 2015). Like in T1DM and T2DM, inflammation plays a central role in DEP. DEP-related disorders including AP, CP, PDAC, and pancreatic surgery all feature local and systemic inflammatory responses (Gukovsky et al., 2013; Sit et al., 2014; Habtezion et al., 2019; Mayerle et al., 2019). Inflammation induced by T-helper in islet has been suggested to contribute to β cell apoptosis and dysfunction during CP (Talukdar et al., 2016). Other studies indicate that pancreatic levels of interferon gamma (IFNγ) are significantly higher in diabetic CP patients and induce β cell dysfunction via Pancreatic Duodenum Homeobox-1 (PDX-1)-downstream signaling pathways (Pavan Kumar et al., 2012; Pondugala et al., 2015). Inflammation can also increase ROS and β cell death by modulating Nrf2/NF-κB and SAPK/JNK pathways (Choudhury et al., 2015). Cytokine macrophage migration inhibitory factors overexpressed in pancreatic cancer are related to impaired insulin release by β cells (Tan et al., 2014). Primary β cell destruction in diabetes associated with autoimmune pancreatitis (AIP) may be induced by T-cells (Tanaka et al., 2001).

Although inflammation is a major factor in DM, the source of inflammation varies depending of the disorder. Inflammation in DEP is mainly induced by exocrine pancreatic damage while autoimmune insulitis is the source of inflammation in T1DM, and long-term insulin resistance and hyperglycemia are the main drivers of chronic inflammation in T2DM. Whether these factors contribute differentially to β cell dysfunction and disease progression in these three DM disorders needs further investigation.

## Role of β Cell Dedifferentiation in DM Disorders

Previous studies have suggested that islet damage and apoptotic β cells do not fully explain the decline in β cell function in T2DM (Rahier et al., 2008; Cinti et al., 2016). There is increasing evidence that mature β cells may, under certain conditions, lose their differentiated phenotype and cell characteristics and degenerate to a less differentiated state. These dedifferentiated cells do not produce insulin, and no longer contribute to glucose homeostasis. So, both dedifferentiation and cell death have been proposed be involved in the damage of β cell mass in diabetes (Bensellam et al., 2018). The detailed mechanism of β-cell dedifferentiation is still unclear. Certain stress related conditions, including inflammation, hypoxia/oxidative stress, and ER stress, may cause β-cell dedifferentiation. Therefore, it is considered that dedifferentiation may be a mechanism for cells to adapt to the environment changes to avoid death. Beta cell dedifferentiation is reversible before the disease progresses to a certain degree. It has been shown that modulating blood glucose to normal levels can lead to the reversal of β cell dedifferentiation.

Talchai et al. (2012) found that β cell dedifferentiation is a critical cause of β cell function reduction in a mouse model of T2DM. Clinical studies further confirmed that β cells can dedifferentiate into α- and δ-“like” cells in patients with T2DM (Jonas et al., 1999; Cinti et al., 2016; Diedisheim et al., 2018). Further investigations are needed to confirm the role of β cell dedifferentiation in damages of β cell functions in T2DM.

Using a pancreatic duct ligation (PDL) model of CP to induce DEP, a recent study showed that the decrease in the number and function of β cells caused by was due to dedifferentiation rather than apoptosis of β cells, and was associated with inflammatory cell infiltration (Xiao et al., 2017). In the islets of CP patients, more than 10% of β cells undergo dedifferentiation, and the dedifferentiation rate of β cells continues to accelerate during the progress of the disease (Sun et al., 2019). These phenomena suggest that the dedifferentiation of β cells may be an important cause of DEP.

## β Cell Replication, Transdifferentiation, and Neogenesis in DM Disorders

In T1DM and T2DM, compensatory mechanisms operate to preserve β cell mass. Studies in various rodent pancreatic islet injury models and samples from children after pancreatectomy have demonstrated the regeneration of β cells (Collombat et al., 2010; Sharma et al., 2017; Shi et al., 2018). Furthermore, increasing β cell mass has been noticed during various clinical conditions, including pregnancy and obesity that can cause islet hyperplasia, as well as in animal models of insulin resistance (Ouyang et al., 2011). Studies of genetic lineage tracing in mice show that replication of pre-existing β cells is the main resource of β cell replenishment, in both healthy and injured conditions. However, it is less clear about how big a role of replication plays in humans (Cerf, 2013).

In addition, one report shows both αand δ cells may transdifferentiate into β cells when more than 99% of β cells in mice are ablated resulting in recovery of β cell mass over several months, depending on the age of the mice (Zhou and Melton, 2018). The molecular mechanism and whether such conversions also occur in humans remain to be explored.

The existence of pancreatic stem cells (or known as progenitor cells) has been suggested. It has been reported that new islet cells could be generated directly from ducts (referred to as neogenesis) (Bonner-Weir et al., 2012). In another study, it was shown that a population of cells with positive expression of neurogenin 3 (Ngn3), indicating the function of these cells as endocrine precursor, appeared around islets and ducts after PDL in experimental animal models (Magenheim et al., 2011). In T2DM, neogenesis but not proliferation was suggested to be a potential mechanism for the increase of β-cell mass in subjects with insulin resistance and impaired glucose tolerance. The existence of adult pancreatic stem cells still needs to be further clarified (Zhou and Melton, 2018).

Several studies have suggested β cell compensation may occur in DEP. For example, in mice model of cerulein-induced pancreatitis, it is possible to generate β cells from cytokeratin 5-positive cells (Shi et al., 2018). Leukemia inhibitory factor induced activation of the Jak/Stat signaling induced non-endocrine pancreatic cells trans-differentiating into insulin-producing cells (Yamauchi et al., 2015). Lineage-tracing studies present evidence that exocrine cells undergo dedifferentiation into a progenitor-like state from which they can be manipulated to form insulin-producing cells (Jawahar et al., 2019). Cells with a positive expression of chromogranin A, but no hormone production [known as chromogranin A positive hormone-negative (CPHN) cells], are highly abundant in neonatal, therefore are considered as candidate of newly formed endocrine cells. The regeneration of endocrine cell has been suggested by the appearance a lobular distribution of these CPHN cells in CP (Moin et al., 2018). It was suggested that a microenvironment suitable for regeneration of acinar- and β cell could be induced by activated macrophages (Criscimanna et al., 2014). Therefore, similar β cell compensatory mechanisms as in T1DM and T2DM may play important role in DEP. However, whether there is a difference in proportions of various compensations and the source of compensatory β cells in different types of DM needs to be determined.

**FIGURE 1:**
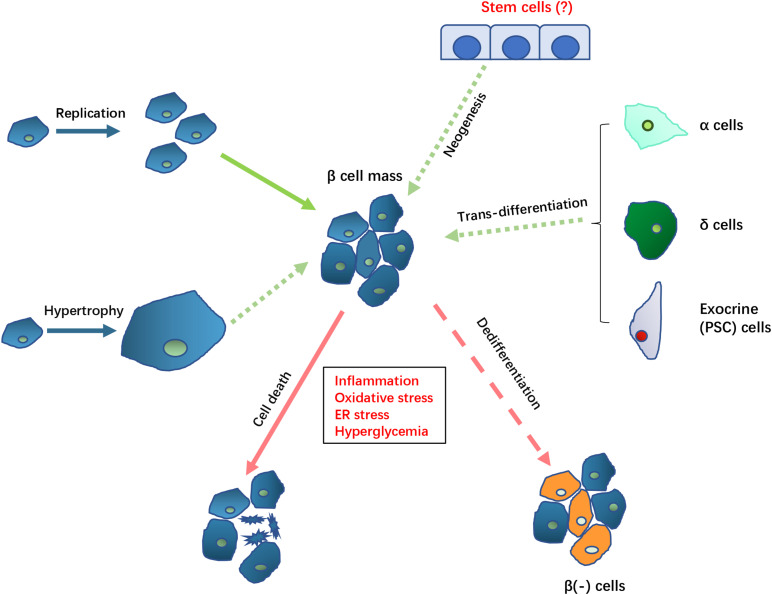
Potential mechanisms controlling β cell mass in DEP. The loss of β cell mass (indicated by arrows with red lines) in DEP could result from cell death and β cell dedifferentiation which causes loss of β cell function and specific markers. The loss of β cell mass may be compensated (indicated by arrows with green lines) by replication or hypertrophy by remaining β cell, trans-differentiation from non-β cells, as well as neogenesis of new β cell from potential existing stem cells. Solid arrows indicate known mechanisms while dotted arrows indicate the potential mechanisms that need to be further clarified in DEP.

The potential mechanisms involved in controlling β cell mass and function are summarized in Figure 1.

## α Cell and Glucagon Response

The α cells of Langerhans islets secrete glucagon, which acts on the hepatocytes to enhance glycogenolysis and gluconeogenesis. In T1DM, reduced cell mass in endocrine islets appears to be highly specific to β cells. It has been reported that three quarters of islet were composed of α cells, and the level of circulating glucagon can be markedly elevated despite hyperglycemia (Gromada et al., 2018). Studies in T1DM have shown that in addition to causing hyperglycemia through increased level of glucagon, α cell dysfunction may also result in insufficient glucagon release in situation of hypoglycemia. Hyperglucagonemia in T1DM is thought to be due to hypertrophy and hyperplasia of α cell. In addition, loss of insulin influence may cause disinhibition of α cells and play a role in hyperglucagonemia (Campbell and Drucker, 2015). T2DM patients are often detected with enduring fasting hyperglucagonemia, as well as insufficient postprandial suppression of glucagon levels, suggesting glucagon as an important contributor to the hyperglycemia of T2DM. Although whether α cell mass is unchanged or increased in patients with T2DM is debatable, the α to β cells ratio is significantly higher in T2DM patients due to the decreased β cell mass (Campbell and Drucker, 2015). It was suggested that in patients with T2DM, α cell mass and glucagon secretion were enhanced by high glucose, lipids, and inflammatory cytokines. Hyperglucagonemia is considered to be caused by α cell resistance to the inhibitions of insulin and hyperglycemia. In addition, dysregulation of incretins, may also be involved since the incretins function as suppressor of glucagon release. α cells have higher resistance to toxicity stimulation of glucose, lipid, and cytokines, which might be potential cause for relatively less affected α cell mass. Impaired glucagon secretion could lead to decreased hepatic glucose production in spite of dangerously low blood glucose levels (hypoglycemia unawareness) (Andersen et al., 2018; Gromada et al., 2018). In addition, impaired α cells severely limits the ability to regulate treatment related hypoglycemia (Lin et al., 2015), which used to be considered a minor problem in T2DM, but is now thought to be underestimated and drawing more attentions due to elevated usage of insulin treatment (Zammitt and Frier, 2005).

Glucose counter regulation was reported to be retained with slightly damage in CP related DM patients, even with no residual β cell function (Larsen et al., 1990). Even when glucagon secretion could not be properly stimulated by insulin-induced hypoglycemia in CP related DM patients, preserved catecholamine response to hypoglycemia largely preserved glucose counterregulation. These findings suggest that in insulin-dependent diabetes mellitus secondary to CP, the adverse response to hypoglycemia may be avoidable. High somatostatin levels may help lower blood sugar levels in CP patients (Larsen et al., 1988). In a study of DM after CP along with T2DM, in both diseases, the response of α cell s to oral glucose intake and hypoglycemia was similarly disrupted, suggesting a common etiology arising secondary to DM development (Mumme et al., 2017). Islet α cell inflammation was suggested to be among the cause of hypoglycemia (Li et al., 2018). In CP patients with low BMI, elevated levels of α cells have been reported to emanate from the pancreatic duct (Webb et al., 2018). As a special type of CP, AIP patients who underwent steroid therapy showed improved pancreatic α cell function but only partial improvement of β cell function (Takeshima et al., 2018). This imbalance between α cell and β cell function may contribute to difficulties in glucose control in DEP.

## Pancreatic Polypeptide (PP) Cells

PP cells are mainly distributed in the pancreas head, with pancreatic polypeptide as their endocrine hormone product (Rahier et al., 1983). Since the β cells are surrounded by PP cells, a protective role was suggested for PP cells. PP can stimulate insulin secretion in isolated islets. The mechanism was suggested to be mainly through the inhibition of somatostatin secretion (Kim et al., 2014). In T2DM, PP levels is elevated, which causing decreased secretion of somatostatin, which may be a compensatory mechanism to regulate glycemia. Experiments in animals showed that infusion of PP could not improve glucose tolerance, neither did it increase the release of insulin. However, PP decreased hepatic glucose production, which may improve hepatic insulin sensitivity (Hennig et al., 2002). These results suggest that PP can reduce the liver’s glucose production by reducing hepatic insulin resistance. Hepatic insulin resistance has been reported in CP, which may be caused by decrease in PP. In addition, damage to the PP receptor stimulation signal of the hepatic cells may also be involved (Sliwinska-Mosson et al., 2017).

Decreased PP production has been suggested to be an earl marker of islet dysfunction. Patients with CP remain at low PP levels which are associated with the severity of the degree of pancreatic lesions. Impairment of postprandial PP response is among the earliest manifestations of pancreatic endocrine abnormalities in CP (Hanafusa et al., 1982). Functional impairment of PP cells and significant loss of PP response can be observed in DEP patients with complete destruction of β cells (Larsen et al., 1988). PP secretion was absent in CP without endogenous insulin production. In AP and CP, PP has been suggested to be used as a diagnostic marker as well as treatment target of DEP (Hennig et al., 2002). However, fasting PP levels are similar in CP, PDAC, and controls regardless of glycemic status (Nagpal et al., 2018), indicating that PP might not be a specific diagnostic marker for DEP. In addition, 58% of patients with CP were reported to be with normal levels of PP, suggesting that PP may be not a sensitive marker for diagnosis of CP (Andersen et al., 1980). PP response to mixed meal may be a better biomarker to help distinguish DEP from other types, a hypothesis that we are currently testing (Hart et al., 2018). Hepatic insulin resistance was reported in DEP patients with preceding CP, PDAC, pancreatectomy and cystic fibrosis (Seymour et al., 1988; Cersosimo et al., 1991; Kien et al., 1995; Brunicardi et al., 1996). CP accompanied by PP deficiency is associated with partial hepatic resistance in both regular condition and situations of hyperinsulinemia (Brunicardi et al., 1996). A previous study showed that PP supplementation can improve hepatic insulin sensitivity and reduce glucose production by the liver (Brunicardi et al., 1996). However, absent PP secretion may not have a major effect on glucoregulation in diabetes secondary to CP (Larsen, 1993).

## δ Cell and Somatostatin

The δ cells secrete somatostatin when glucose is elevated. Once released, somatostatin functions on islet cells through its receptor (SSTRs) as a local paracrine agent. Somatostatin has a large number of effects on α and β cells, which together leads to a decrease in secretion of insulin and glucagon. Somatostatin also have influences on β cell. It can modulate the apoptosis and proliferation of mouse β cells in response to stress (Damsteegt et al., 2019). In addition, δ cell can direct transdifferentiate β cell therefore plays a role in β cell mass. It has been reported that in diabetes, restoration of β cells could result from reprogramming of pancreatic δ cells into insulin producers (Chera et al., 2014). The latter results are consistent with the hypothesis of cell plasticity. Transdifferentiation between α, β, and δ cells may be an important mechanism for the regulation of islet function and participate in physiological and pathological processes. How big a role this cell plasticity, and somatostatin regulation plays in human remains to be further studied (Rorsman and Huising, 2018).

Although the importance of somatostatin in both diabetes and pancreatic disease have been realized, its role in DEP settings has not been thoroughly studied. It has been previously reported that plasma somatostatin levels increased in patients with insulin-dependent DM after CP. The source of these somatostatins is still need to be clarified. However, it is known that somatostatin may help lower blood glucose in patients with insufficient endogenous insulin secretion by inhibiting glucagon secretion (Larsen, 1993). One of the functions of somatostatin is to regulate GLP-1 release. It has been reported that the GLP-1 response seems intact in DEP, while the response to glucose-dependent insulinotropic polypeptide (GIP) is reduced, similarly, as in T2DM (Hedetoft et al., 2000; Knop et al., 2007a). Postprandial secretion of GLP-1 is significantly elevated after trypsin replacement therapy in CP patients (Knop et al., 2007c).

## ε Cell and Ghrelin

ε cell was the fifth endocrine cells identified in pancreatic islet cells, which producing the ghrelin hormone (Wierup et al., 2002). Ghrelin functions through regulating the functions of other endocrine cells (Sakata et al., 2019). Ghrelin inhibits the secretion of insulin from β cells via paracrine interaction between δ cells and β cells. Transcriptome analysis showed that ghrelin had specificity in activating δ cells to enhance somatostatin release (DiGruccio et al., 2016). It also stimulates appetite and growth hormone secretion. In addition, ghrelin also plays a role in regulating pancreatic development and physiology, participates in regulating the proliferation and vitality of β cells. In addition, it can also play a role in inhibiting endocrine and pancreatic acinar function. Ghrelin is currently an important research target in T2DM and obesity, and its multiple roles in energy metabolism have received widespread attention (Yada et al., 2014; Churm et al., 2017; Napolitano et al., 2018). The role of ε cells and ghrelin in DEP has not been well documented. Giving the multiple functions it exerts in pancreatic function and nutritional status, its involvement in the pathogenesis of DEP need to be further explored.

Damage and dysfunction of the endocrine islet cells are important mechanisms for DEP pathogenesis. Both insulin and glucagon levels are decreased due to the destruction of β and α cells, accompanied with decreased PP and increased somatostatin levels. The microenvironment stimulation and signaling in the pancreatic islet may affect proliferation of β cell (Aamodt and Powers, 2017). A perspective from multiple intra-islet cell interconversion may facilitate a more thorough understanding of the endocrine pathological mechanism of DEP.

**FIGURE 2:**
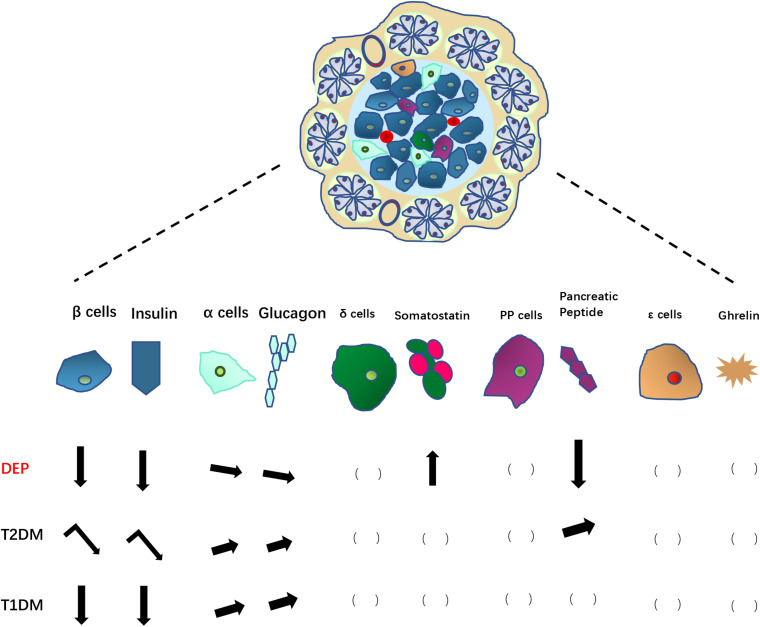
Changes in pancreatic endocrine cells and associated hormones in DEP, compared to those in T2DM and T1DM. Arrows pointing down indicate decreases in number of endocrine cells or hormone levels; Arrows pointing up indicate increases in number of endocrine cells or hormone levels; leaning arrows indicate slight increase/decrease; turning arrows indicate a two-step changes (increase first then decrease). The blank brackets indicate unknown situation need to be further studied.

The overall changes of endocrine pancreatic islet cells and hormones are summarized in Figure 2.

## Exocrine Pancreas: Pancreatic Stellate Cells (PSCS)

Acinar cells and duct cells are the major components of exocrine pancreas. The pathogenetic changes of these cells may have influences on the structure and functions of endocrine islet as well. Activated PSCs play a central role in regulating extracellular matrix (ECM) protein synthesis and degradation (Omary et al., 2007; Apte et al., 2011) and was suggested to be correlated with occurrence and progression of CP and pancreatic cancer (Apte et al., 2015). PSCs also secrete various cytokines (Xue et al., 2018), which cause β cell dysfunction (Zang et al., 2015), leading to hyperglycemia, further aggravating the adverse effects of PSCs on β cells (Zechner et al., 2014; Zha et al., 2014). PSCs-induced β cell dysfunction can be summarized as three components: (1) physical destruction of islets caused by a large number of ECM, such as collagen I and fibronectin, resulting in pancreatic fibrosis, which leads to islet dysfunction (Sasikala et al., 2012; Hart et al., 2016); (2) pathogenic factors such as inflammatory cytokines, oxidative stress and subsequent inflammatory response lead to islet dysfunction; (3) potential role of exosomes secreted by PSCs. In addition, although the relevant experimental evidence is still insufficient, studies have found that PSCs have characteristics of stem/progenitor cells, suggesting that PSCs may have potential in pancreatic and islets regeneration and thus could play an important role in treatment of DEP in the future (Apte et al., 1998; Trim et al., 2000; Lardon et al., 2002; Joanette et al., 2004).

## Insulin Resistance

In the past, insulin resistance was considered to be an unimportant contributor to DEP related to CP (Kumar et al., 2017). However, changes in insulin sensitivity have been documented in CP (Vlasakova et al., 2002), and DEP caused by total pancreatectomy (Yki-Jarvinen et al., 1986). One study of 30 patients with CP (4 normal glucose tolerant, 4 impaired glucose tolerant, 22 diabetic) found insulin resistance was present in 22 of the 30 patients and was more common in those with impaired glucose tolerance (IGT) (3 of 4) and diabetes (17 of 22) (Niebisz-Cieslak and Karnafel, 2010). A physiologic study of preoperative patients with pancreatic cancer also found higher insulin resistance compared to matched controls (Cersosimo et al., 1991). Traditional T2DM risk factors for insulin resistance such as family history, obesity was also important in DM development in CP patients (Bellin et al., 2017). In fibrocalcific pancreatic diabetes (FCPD), although insulin secretion defects are considered major cause, the potential involvement of insulin resistance in pathogenesis has been more oftenly recognized (Dasgupta et al., 2015; Aiswarya et al., 2019). In AP, DEP was more frequently present in patients with higher disease severity of AP (Vujasinovic et al., 2014), and insulin resistance index was higher among these patients (Wu et al., 2011). In pancreatic cancer-associated diabetes, Galectin-3 and S100A9, which are related to DM development were reported to mediate insulin resistance (Liao et al., 2019).

## Potential DEP Pathological Mechanisms Based on Pancreatic Exocrine Insufficiency (PEI)

Pancreatic diseases are often accompanied with PEI and malnutrition. In AP, prevalence of PEI during admission was 62 and 21–35% during follow up (Vujasinovic et al., 2014; Tu et al., 2017; Huang et al., 2019). After pancreatectomy, PEI was reported to develop in 36–76% of the patients with mean time to onset of 14–40 months (Lee et al., 2013; Beger et al., 2018; Hallac et al., 2019; Kusakabe et al., 2019). PEI facilitates pathology of DEP through malnutrition, regulation of incretin secretion, and crosstalk with intestinal flora.

## Influence of PEI and Malnutrition

It is well known that CP patients are often with deficient nourishment due to malabsorption and increased metabolic activity (Shimosegawa, 2019). For example, CP patients often have decreased lean body mass and fat mass, which could lead to decreased functional capacity and further weight loss (Gilliland et al., 2017). The causes of CP nutritional deficiencies are multifaceted, including exocrine and/or endocrine dysfunction, severe abdominal pain, etc. leading to less food intake, often persistent alcohol consumption, and increased metabolic activity (O’Brien and Omer, 2019). The malnutrition severity was reported to be correlated with malabsorption and nutrients depletion, as well as increased metabolic activity (Rasmussen et al., 2013).

Pancreatic disease has differential effects on various nutrient components. In PEI, carbohydrate digestion is maintained, protein digestion is mildly impaired, while lipid digestion is most significantly impaired. Digestion of protein mainly depend on proteolytic activity in the stomach rarely damaged in CP. Lipids are mainly digested in the small intestine, and pancreatic lipase and coenzyme play a key role in this process. Due to the dysfunction of lipid digestion in CP patients, deficiency of fat-soluble vitamins A, D, E, and K are common. In addition, insufficient secretion of pancreatic protease may cause vitamin B12 deficiency. Mineral intake and absorption are also affected in pancreatic disease (Papazachariou et al., 2000; Vujasinovic et al., 2019). Several markers change significantly in patients with PEI, including albumin, phosphorus, and fat-soluble vitamins (Alexandre-Heymann et al., 2019). Reductions of plasma amino acid levels have also been seen in CP, particularly sulfur containing amino acids and branched chain amino acids (Girish et al., 2011).

Changes of nutrient substrates may play a role in DM development. For example, various free fatty acids (FFA) have may cause various effects on different physiological processes, such as changing the formation and decomposition of adipose tissue, resulting in corresponding changes in endocrine and inflammatory responses. It may also cause changes in cell membrane composition. These effects of plasma FFA may eventually lead to insulin resistance (Sobczak et al., 2019). High levels of saturated FFA have been suggested to be correlated with impaired glucose tolerance in both T1DM and T2DM (Maulucci et al., 2016). Diacylglycerol signaling pathway regulates pancreatic β cells and insulin secretion (Kaneko and Ishikawa, 2015). Ceramide also plays a critical role in DM development (Galadari et al., 2013). The relation between nutritional substrates and diabetes has mostly been studied at level of vitamin deficiency. The correlation with disease risk and role in glycemic control of vitamin D in T2DM has been widely studied by many researchers including us (Wu et al., 2017; Munoz-Garach et al., 2019). In addition, mineral and amino acid levels appear to influence the risk of diabetes development. As a secondary disease of pancreas injury, in the study of DEP, attention needs to be paid to the role of malnutrition and deficiency of various substrates.

## Incretin: Gut-Islet Hormone Interaction

Gut-islet interactions, mainly through incretin hormones has been widely studied. GLP-1 is mainly produced by L cells in the ileum and large intestine, while K cells are the main sources of GIP (Holst et al., 2009). During food intake, incretin hormones are released by these intestinal endocrine cells to facilitate insulin secretion response to glucose. Most of postprandial insulin secretion are induced by incretins. Then the rapid degradation of incretins by dipeptidyl peptidase IV (DPP-IV) ensures a transient response. Studies have shown a consistent correlation of the concentration of these peptide hormones with glycemia. In T2DM, GLP-1 secretion appears to be deficient while there appears to resistance to GIP (Nauck et al., 1993). Incretin-based pharmacotherapies (GLP-1 receptor agonists and DPP-IV inhibitors) have become popular choices in clinical treatment of diabetes. However, rather than the main cause leading to T2DM, the deficiency of incretin was more often considered as a results of deteriorating glucose homeostasis in T2DM (Knop et al., 2007b; Knop, 2010). Incretin-based pharmacotherapy has been suggested to be correlated with increased risk of AP in T2DM patients (Azoulay et al., 2016; Ueberberg et al., 2016; Tseng et al., 2017) although more recent and thorough studies have suggested that incretin-based treatment does not increase pancreatitis risk (Wang et al., 2015, 2018; Abd El Aziz et al., 2020). The GLP-1 receptor may activate PSCs, changes pancreatic gene, and enhances pancreatic mass, therefore inducing pancreatic injury (Koehler et al., 2009; Yang et al., 2013).

In normal conditions, the release of incretin hormones is mainly induced by fatty acids and other nutrients. Thus, deficiency in pancreatic exocrine function, which causes impaired fat digestion, may result in impaired incretin response, causing adverse effects on insulin release and blood glucose control. In addition, as discussed above, in settings of pancreatic disease and DEP, damage to δ cells and somatostatin may affect development of DM though influence on incretin hormone response. Some studies suggested that in DEP, patients are still sensitive to GLP-1, while GIP induced insulin secretion response is damaged, similar as in T2DM (Hedetoft et al., 2000; Knop et al., 2007a). However, in CP patients with normal glucose tolerance, the effect of intestinal incretin was preserved, while in patients with secondary DM, the effect of incretin was strongly reduced, indicating that incretin deficiency is the result of diabetes rather than pancreatitis (Knop et al., 2007b). In contrast, a different study in CP with or without diabetes found reduced GIP responses to a test meal in both groups, with no correlation with exocrine insufficiency (Gomez-Cerezo et al., 1996). Differing results between studies may reflect the meal tested; a study in CP found similar GIP response as controls to a mixed meal but a reduced response to a 100% fat meal (Ebert and Creutzfeldt, 1980).

Incretin functions in regulating survival, cell growth and differentiation of pancreatic cells which may play a role in β cell restoration and genesis (Tasyurek et al., 2014). For example, pueraria tuberosa tubers (PTY-2), which acts as an incretin receptor agonist, has been shown to inhibit β cell apoptosis therefore protects streptozocin (STZ)-induced diabetes (Srivastava et al., 2018). GLP-1 and gastrin signaling induce *in vivo* reprogramming of pancreatic exocrine cells into β cells (Sasaki et al., 2015). Considering the complicated effects of incretins on exocrine function deterioration and potential β cell protection, their roles in DEP pathogenesis, as well as the choice of incretin-based therapy in these patients need more careful studies.

## Organ Crosstalk: Intestinal Microbiota

The gastrointestinal microbiota is an important physiological factor that has emerged in recent years. The composition of the gut microbiota is affected by a number of factors, including diet, disease state, drugs, and host inheritance (Torres-Fuentes et al., 2017). Changes in the composition of intestinal microbiota, which exert regulatory functions on metabolism and inflammation through various organs (Armutcu, 2019; Tilg et al., 2019). Microbial imbalances (also known as dysbacteriosis) are associated with immune effector cells dysregulation, as well as the levels of inflammatory cytokines, therefore are considered an important factor in different inflammation- mediated diseases (Memba et al., 2017).

A disturbed intraduodenal milieu and pancreatic damage in advanced CP may lead to changes in the intestinal microbiota. Impaired intestinal mucosal barrier integrity plays a critical role in microbiota changes. The changes in intestinal ecological system and bacterial metabolism may in turn affect diabetes and metabolic abnormalities (Jandhyala et al., 2017). Therefore, it is a possibility that gut microbiota might play an important role in DEP. There has been already a small number of reports on intestinal microbiota in DEP, especially which secondary to CP. The most recent study in India enrolled healthy control, CP patient, and DEP patients secondary to CP. Significant differences in the abundance of certain bacteria species, including *phylum Bacteroidetes* and *Faecalibacterium* were identified among the three groups (Jandhyala et al., 2017). A reduction in the abundance of *Faecalibacteriumprausnitzii* and increase in plasma endotoxin were observed in non-diabetic CP, which was more pronounce in CP with diabetes. There was a significant negative correlation between fasting and postprandial blood glucose with the abundance of *Faecalibacteriumprausnitzii*, and a positive correlation with plasma insulin levels with bacteria, suggesting that intestinal microbial disorders are associated with metabolic changes in CP.

**FIGURE 3:**
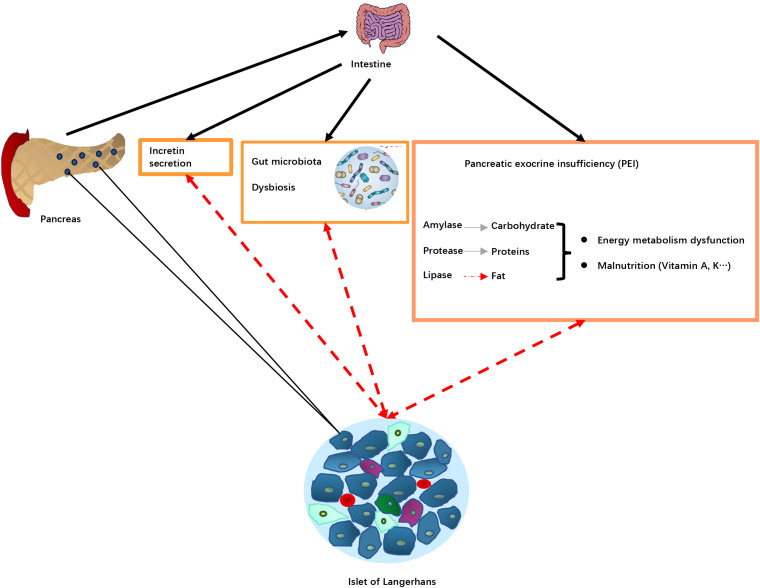
Potential pathological mechanisms associated with pancreatic exocrine insufficiency (PEI) in DEP. Pancreas damage in DEP leading diseases results in reduced release of digestive enzymes into the intestine, that in turn leads to PEI, decreased food digestion and malnutrition. PEI may also affect incretin secretion and the gut microbiota resulting in dysbiosis. These changes alter islet of Langerhans function (dotted red arrows), resulting in changes in production and release of hormones involved in blood glucose regulation. Pancreas damage in DEP leading diseases results in reduced release of digestive enzymes into intestine and impaired nutrient digestion, causing PEI. Lipid digestion is the most significantly affected, which in turn can cause deficiency of fat-soluble vitamins, as well as intake of some minerals. The malnutrition status may play a role in DM development. For example, disturbed plasma lipid profiles may lead to insulin resistance, and certain vitamin deficiency could increase risk of insufficient glycemic control. In addition, PEI and impaired fat digestion can result in impaired release of incretin hormones, glucagon-like peptide-1 (GLP-1) and glucose-dependent insulinotropic polypeptide (GIP), which are the main regulator of insulin release and blood glucose control after meal, as well as regulator of β cell mass through regulation of survival and differentiation of multiple types of pancreatic endocrine cells. Furthermore, a disturbed intraduodenal milieu and pancreatic damage in advanced CP may lead to changes in the intestinal microbiota. The changes in intestinal ecological system and bacterial metabolism may in turn affect diabetes and metabolic abnormalities.

The potential pathological mechanisms of DEP through the influence of PEI are summarized in Figure 3.

## Summary

Due to the lack of consensus diagnostic criteria and experimental animal models, our knowledge of the pathophysiologic mechanisms underlying DEP is still at a very early stage. DEP is characterized by prior pancreatic disease especially AP and CP. Therefore, damage in the whole pancreas, including all endocrine cells and exocrine pancreas, as well as interactions among these cells, need to be considered when exploring DM development.

Physiologically, DEP patients exhibit significant reduced β cell functions, including both β cell mass and insulin secretion capability. In addition to the direct cell death due to the stress environment in pancreatic disease, other mechanisms involved in β cell mass and function control, such as dedifferentiation, transdifferentiation and neogenesis need to be further explored. The interchangeable features of different endocrine cells highlight the need to carefully quantify different types of cells in DEP.

Alpha cells appear to be less impaired in DEP, however, the glucagon response could be compromised due to increased somatostatin. The imbalance between β and α cells may also contribute to glycemic control difficulties in DEP.

PP plasma levels were reduced in CP, especially in CP with DM. However, it seems to be a result of higher sensitivity of δ cells to inflammation damage and PP deficiency does not appear to play a critical role in DM development.

PEI is common in DEP. PEI may affect DM development through malnutrition, dysregulation of incretins that play a critical role in glucose homeostasis, and cross-talk with the intestinal microbiota.

Considering all of the above information, we suggest that studies on pathological mechanisms of DEP should concentrate on the following directions:

(i) In clinical settings, the priority is to establish the diagnosis standard and screening procedure of DEP. A clearly defined and easily applicable diagnostic criterion should precede all effective clinical studies of DEP pathological mechanism.
(ii) β cell mass and function regulation, especially mechanisms related to inflammation regulation should be the most important pathological mechanism considered. We suggest the priority in DEP studies should be to clarify the threshold of β cell mass that causes hyperglycemia, as well as inflammatory cytokines and immune cell infiltration in the endocrine islet.
(iii) The involvement of other pancreatic cells (mainly endocrine islet cells) in DM pathology should be given more attention. Clarification of the detailed compositions of cell types in islets during DEP disease development will provide substantial information on the pathological progress.
(iv) PEI related mechanisms in DEP should be further elucidated. The level and changes of intestinal hormones, compositions of intestinal microbiota as well as their metabolites will provide further knowledge of systemic causes of DEP.
(v) In basic research of DEP pathological mechanisms, experimental systems, both animal models and co-culture cell system are warranted. On the cellular level, dedifferentiation, transdifferentiation and neogenesis of β cells, especially from other endocrine and exocrine pancreatic cells are of great importance.

It should be kept in mind that DEP, although different from T1DM and T2DM in clinical features, may possess similar basic pathological mechanisms when considered at tissue and cellular levels. In T1DM and T2DM, the risk of pancreatitis is elevated. As systemic diseases originate from damage/dysfunction in pancreatic islets, the differences between different types of DMs may be subtle. DEP with different preceding pancreatic diseases may show different similarities to T1DM and T2DM. For example, a recent study comparing genetic risks between diabetes associated with CP (CP-DM) and T2DM found that the two diseases have similar characteristics in single nucleotide polymorphisms, suggesting that CP-DM may be a subtype of T2DM (Goodarzi et al., 2019). Further exploration of pathological mechanisms, besides application in optimization of clinical management, may results in new classifications of the types of DM in the future. It should be pointed out that DEP is comprised of several distinct diabetes subtypes such as PPDM-A (post-acute pancreatitis diabetes mellitus), PPDM-C (post chronic pancreatitis diabetes mellitus), PCRD (pancreatic cancer-related diabetes), and CFRD (cystic fibrosis-related diabetes) (Petrov, 2017). The literature reviewed in this article were mainly in PPDM, especially PPDM-C. Since different subtypes of DEP seem to have different underlying pathophysiological mechanisms, specific attention should be paid in studies of other subtypes of DEP.

## Author Contributions

QW and LQ wrote the first draft. HL, DL, XZ, and YD researched data for the review. LL, SP, MG, AL, and RW edited the manuscript before submission. All authors read and approved the final version of the manuscript.

## Conflict of Interest

The authors declare that the research was conducted in the absence of any commercial or financial relationships that could be construed as a potential conflict of interest.
